# Dr. Levi Cooper Lane

**Published:** 1902-03

**Authors:** 


Dr. Levi Cooper Lane, in San Francisco, the eminent surgeon, died at his home February 19, 1902, aged 69 years. His death was due to a general breakdown of the system. He was the founder of the Cooper Medical College and the Lane Hospital.

